# Development and Evaluation of a Novel Protein-Based Assay for Specific Detection of KPC β-Lactamases from Klebsiella pneumoniae Clinical Isolates

**DOI:** 10.1128/mSphere.00918-19

**Published:** 2020-01-08

**Authors:** Shuo Lu, Victoria Soeung, Hoang A. T. Nguyen, S. Wesley Long, James M. Musser, Timothy Palzkill

**Affiliations:** a Department of Pharmacology and Chemical Biology, Baylor College of Medicine, Houston, Texas, USA; b Department of Pathology and Genomic Medicine, Houston Methodist Hospital Research Institute, Houston, Texas, USA; c Department of Pathology and Laboratory Medicine, Weill Cornell Medical College, New York, New York, USA; d Department of Biochemistry and Molecular Biology, Baylor College of Medicine, Houston, Texas, USA; JMI Laboratories

**Keywords:** antibiotic resistance, beta-lactamase, carbapenemase, diagnostic, protein engineering, sensor

## Abstract

Carbapenemases confer resistance to nearly all β-lactam antibiotics. The extensive spread of carbapenemase-producing multidrug-resistant bacteria contributes significantly to hospital-acquired infections. We have developed a novel protein-based binding assay that identifies KPC β-lactamases from clinical isolates. We used the protein-protein interaction between KPCs and a soluble β-lactamase inhibitory protein (BLIP) variant, BLIP^K74T/W112D^, which specifically inhibits KPCs but not other β-lactamases. In this assay, BLIP^K74T/W112D^ was allowed to form complexes with KPC-2 in bacterial cell lysates and then extracted using His tag binding resins. We demonstrated the presence of KPC-2 by monitoring the hydrolysis of a colorimetric β-lactam substrate. Also, to further increase the accuracy of the method, a BLIP^K74T/W112D^-mediated inhibition assay was developed. The binding and inhibition assays were validated by testing 127 Klebsiella pneumoniae clinical isolates with known genome sequences for the presence of KPC. Our assays identified a total of 32 strains as KPC-2 producers, a result in 100% concordance with genome sequencing predictions. To further simplify the assay and decrease the time to obtain results, the BLIP^K74T/W112D^ protein was tested in combination with the widely used Carba-NP assay. For this purpose, the genome-sequenced K. pneumoniae strains were tested for the presence of carbapenemases with the Carba-NP test with and without the addition of BLIP^K74T/W122D^. The test accurately identified carbapenemase-producing strains and the addition of BLIP^K74T/W112D^ allowed a further determination that the strains contain KPC carbapenemase. Thus, the BLIP^K74T/W112D^ protein is an effective sensor to specifically detect KPC β-lactamases produced by clinical isolates.

**IMPORTANCE** Infections caused by carbapenem-resistant *Enterobacteriaceae* are associated with high therapeutic failure and mortality rates. Thus, it is critical to rapidly identify clinical isolates expressing KPC β-lactamases to facilitate administration of the correct antibiotic treatment and initiate infection control strategies. To address this problem, we developed a protein-based, KPC-specific binding assay in combination with a cell lysate inhibition assay that provided a 100% identification rate of KPC from clinical isolates of known genomic sequence. In addition, this protein sensor was adapted to the Carba-NP assay to provide a rapid strategy to detect KPC-producing isolates that will facilitate informed treatment of critically ill patients.

## Abstract (precis)

Infections caused by carbapenem-resistant *Enterobacteriaceae* are associated with high therapeutic failure and mortality rates. Thus, it is critical to rapidly identify clinical isolates expressing KPC β-lactamases to facilitate administration of the correct antibiotic treatment and initiate infection control strategies. To address this problem, we developed a protein-based, KPC-specific binding assay in combination with a cell lysate inhibition assay that provided a 100% identification rate of KPC from clinical isolates of known genomic sequence. In addition, this protein sensor was adapted to the Carba-NP assay to provide a rapid strategy to detect KPC-producing isolates that will facilitate informed treatment of critically ill patients.

## INTRODUCTION

β-Lactams are the most often used antimicrobials in medicine and account for 65% of all antibiotic prescriptions in the United States (1). β-Lactam antibiotics work by inhibiting cell wall biosynthesis in bacteria. There are four primary mechanisms by which bacterial pathogens overcome these antibiotics: production of β-lactamase enzymes, alteration of the active sites of penicillin binding proteins (PBPs), decreased expression of outer membrane proteins (OMPs), and expression of multidrug efflux pumps (2). The most common resistance mechanism is the production of β-lactamases, enzymes that inactivate drugs by hydrolyzing the β-lactam ring (3). β-Lactamases are broadly classified as serine hydrolases that use a nucleophilic serine residue or metalloenzymes that use Zn^2+^ ions in the active site to facilitate substrate hydrolysis (4). Currently, >2,800 naturally occurring β-lactamases have been identified, and each has a unique amino acid sequence and characteristic hydrolysis profile for different β-lactam antibiotics (3, 5).

Carbapenems are an important class of β-lactam antibiotics because they have a broad spectrum of antibacterial activity and are resistant to the action of most β-lactamases (6). Because of these properties, they are considered the last defense for treatment of bacterial infections (6). The extensive use of carbapenems, however, has resulted in the emergence of β-lactamases that efficiently hydrolyze these antimicrobial agents (7). These β-lactamases, termed carbapenemases, hydrolyze nearly all β-lactams (8), thereby creating a serious public health threat (9). The KPC, NDM-1, and OXA-48 carbapenemases, in particular, have become widespread sources of resistance (7). Of these carbapenemases, the KPC carbapenemases are the most prevalent in the United States and comprise greater than 95% of carbapenemases in *Enterobacteriaceae* strains (10, 11). KPC-2 was first identified in North Carolina in 1997 and has subsequently spread worldwide (7, 12). Infections caused by KPC producers in Klebsiella pneumoniae are associated with high therapeutic failure and mortality rates (9, 13). The KPC-2 enzyme has a broad substrate specificity and efficiently hydrolyzes carbapenems in addition to penicillins and cephalosporins and is also only weakly inhibited by clavulanic acid (8). New β-lactamase inhibitors such as avibactam and vaborbactam have recently been introduced, however, that inhibit KPC and have expanded treatment options (14). Nevertheless, KPC variants have been identified in both laboratory and clinical isolates that show phenotypic resistance to the ceftazidime-avibactam combination for the treatment of serious infections caused by carbapenem-resistant organisms (15, 16).

It is important to rapidly identify clinical isolates that produce carbapenemases in order to inform treatment options and to implement infection control measures to limit their spread (13). Several phenotypic detection methods have been described, including the modified Hodge test (17), the modified carbapenem inactivation method (18, 19), and methods detecting the carbapenemase-mediated hydrolysis products of carbapenems, including the Carba-NP assay (20) and matrix-assisted laser desorption ionization−time of flight (MALDI-TOF) mass spectrometry (13). These methods are rapid and useful but do not identify the specific carbapenemase being produced by the resistant organism. Such information is useful in that antibiotics such as ceftazidime-avibactam have activity against KPC but not metallo-β-lactamases such as NDM, VIM, and IMP. Other tests such as lateral flow immunoassays using antibodies directed against specific carbapenemases (21) or PCR- and DNA sequencing-based methods can detect the specific enzyme produced (22), which may facilitate a more targeted treatment strategy. Here, we describe an additional, highly accurate approach for determining the presence of the KPC carbapenemase that is based on the binding and inhibitory properties of an engineered version of the β-lactamase inhibitory protein (23).

**FIG 1:**
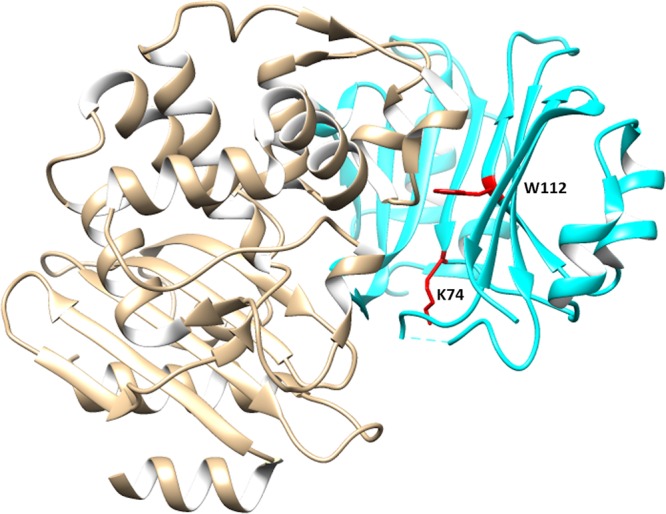
Crystal structures of the KPC-2 and BLIP^wt^ complex (PDB identifier [ID] 3E2L). KPC-2 and wild-type BLIP (BLIP^wt^) are presented in tan and cyan, respectively. Amino acid residues Lys74 and Trp112 in BLIP^wt^ are highlighted in red.

The β-lactamase inhibitory protein (BLIP), originally isolated from Streptomyces clavuligerus, was the first experimentally characterized β-lactamase inhibitory protein (24, 25). BLIP is a 165-amino-acid protein that binds and inhibits the KPC-2 carbapenemase with subnanomolar affinity (26). BLIP also binds to other β-lactamases made by Gram-negative and Gram-positive bacteria with *K_i_* values ranging from picomolar to micromolar (25, 27). The interaction between BLIP and β-lactamases has been used as a model system to provide insight into the determinants of molecular recognition in protein-protein interactions (28, 29). The molecular interface of the BLIP−β-lactamase complex has been extensively studied using structural, computational, and biochemical approaches (26, 29, 30). Additionally, alanine-scanning mutagenesis has been used to identify the amino acid sequence requirements for the binding of BLIP to β-lactamases such as TEM-1, SHV-1, Bla1, and KPC-2 (27, 28, 31). These studies have identified several BLIP amino acid positions that control the specificity of BLIP toward different β-lactamases (Fig. 1) (23, 27, 31). This has made it possible to engineer BLIP as a sensor to identify specific β-lactamases (23).

Previously, we showed that an engineered BLIP^K74T/W112D^ mutant has high specificity for binding KPC-2 but not other β-lactamases (Fig. 1) (23). In addition, it was shown that BLIP^K74T/W112D^-conjugated beads can isolate KPC-2 from crude cell lysates prepared from Escherichia coli laboratory strains expressing KPC-2 and from K. pneumoniae clinical isolates (23). We previously quantitated the presence of KPC-2 by hydrolysis of the colorimetric substrate nitrocefin (23). However, binding conditions were not optimized to enhance signal and reduce nonspecific binding. In addition, only five clinical isolates were tested to validate the specificity of the binding assay, an insufficient number to adequately assess test performance. Finally, we had shown that the BLIP^K74T/W112D^-based detection assay can accurately identify KPC-2, but it was not determined whether the assay could detect other clinically relevant KPC variants such as KPC-3. In the present study, we optimized and expanded the conditions of the assay to identify the most clinically relevant variants of KPC-2. Additionally, we tested the optimized assay against 127 K. pneumoniae clinical isolates and accurately identified all KPC producers, a result 100% concordant with whole-genome sequence predictions. Finally, to improve ease of use, we adapted the BLIP^K74T/W112D^ protein to the well-established Carba-NP assay to allow detection of carbapenemases in clinical strains and determine whether KPC is present.

## RESULTS

### BLIP^K74T/W112D^ potently binds to and inhibits KPC β-lactamase variants.

**FIG 2:**
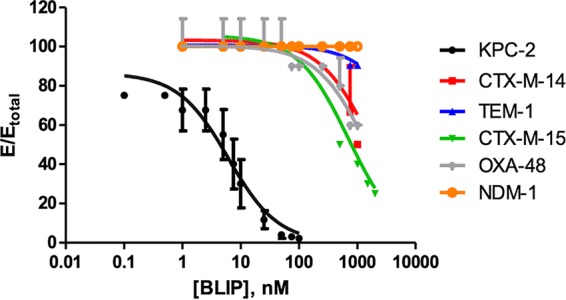
Inhibition curves of BLIP^K74T/W112D^ against KPC-2, CTX-M-14, CTX-M-15, TEM-1, NDM-1, and OXA-48 β-lactamases. The β-lactamases were each mixed with increasing concentrations of BLIP^K74T/W112D^, and initial hydrolysis rates of nitrocefin were determined to assess inhibition. The initial hydrolysis rates are plotted as a function of BLIP^K74T/W112D^ concentration. The fitting curve for determining the *K_i_* is shown as a solid line.

Currently, a total of 39 KPC carbapenemases have been identified based on the U.S. National Library of Medicine database (National Center for Biotechnology Information). However, KPC-2 and KPC-3 are the most prevalent KPC variants found worldwide and are typically responsible for hospital outbreaks (7, 32, 33). In a previous study, we used a mutagenesis and genetic screening approach to identify a BLIP variant (BLIP^K74T/W112D^) that bound to and inhibited KPC-2 but not other class A β-lactamases (23). We tested the hypothesis that the tight binding property of BLIP^K74T/W112D^ is not limited to KPC-2 but instead extends to other variant KPC β-lactamases. We determined the inhibitory constant *K_i_* values of BLIP^K74T/W112D^ toward KPC-2 through KPC-9, CTX-M-14, CTX-M-15, NDM-1, OXA-48, and TEM-1. BLIP^K74T/W112D^ has a *K_i_* value of 2.4 nM toward KPC-2 (Fig. 2 and Table 1). Despite amino acid substitutions in and near the active site, KPC variants 3 through 9 were inhibited by BLIP^K74T/W112D^ with a potency comparable to that of KPC-2 (Table 1), strongly suggesting that BLIP^K74T/W112D^ is a general KPC inhibitor. In agreement with previous data, BLIP^K74T/W112D^ bound weakly to other class A β-lactamases, including the common TEM-1, CTX-M-14, and CTX-M-15 enzymes (Fig. 2 and Table 1) (23). Finally, BLIP^K74T/W112D^ did not inhibit the class B NDM-1 metallo- β-lactamase and very weakly inhibited the OXA-48 carbapenemase (Fig. 2). In the aggregate, these results suggest that the BLIP^K74T/W112D^ mutant is a suitable candidate for developing a protein-based assay to specifically identify KPCs among various β-lactamases.

**TABLE 1:** Inhibition constant *K_i_* values for purified BLIP^K74T/W112D^ variant against several β-lactamases

β-Lactamase	*K_i_* (nM)
KPC-2	2.4 ± 0.4
KPC-3	2.5 ± 0.4
KPC-4	1.2 ± 0.2
KPC-5	1.5 ± 0.3
KPC-6	1.3 ± 0.3
KPC-7	1.5 ± 0.3
KPC-8	1.6 ± 0.3
KPC-9	0.7 ± 0.1
CTX-M-14	908 ± 24
CTX-M-15	433 ± 87
TEM-1	6,240 ± 1,112
NDM-1	NM^*a*^
OXA-48	1,181 ± 129

a NM, no measurable inhibition.

### Development of an *in vitro* KPC β-lactamase-specific binding assay with BLIP^K74T/W112D^.

To establish an *in vitro* assay with high specificity for binding KPC β-lactamases, we optimized our prior assay conditions with various BLIP^K74T/W112D^ concentrations and used a different protein binding strategy compared to our previous study (23). KPC-2 was used as a model protein for the study because BLIP^K74T/W112D^ has a similar affinity for KPC-2 and KPC variants.

To further optimize and validate our approach, we used E. coli strains TP112, TP159, and TP160 expressing the TEM-1, CTX-M-14, and KPC-2 enzymes, respectively. In our previous study, we covalently immobilized BLIP^K74T/W112D^ onto an agarose resin, which could result in heterogeneity of BLIP immobilization and various binding affinities for KPC (23). Hence, rather than covalently immobilizing BLIP^K74T/W112D^ onto a resin, we added various concentrations of BLIP^K74T/W112D^ ranging from 50 to 2,085 nM to bacterial cell lysates and allowed formation of BLIP−β-lactamase complexes to occur in solution. This step maximized the opportunity for native BLIP^K74T/W112D^ to interact with β-lactamases in cell lysates and provided an estimation of the minimum amount of BLIP^K74T/W112D^ required in the assay. Free BLIP^K74T/W112D^ and BLIP^K74T/W112D^/KPC-2 complexes were recovered from cell lysates through binding of the N-terminal His tag on BLIP^K74T/W112D^ to a metal affinity resin. This two-step binding procedure ensures an efficient BLIP^K74T/W112D^ binding interaction with KPC-2 and specific extraction of the complexes.

**FIG 3:**
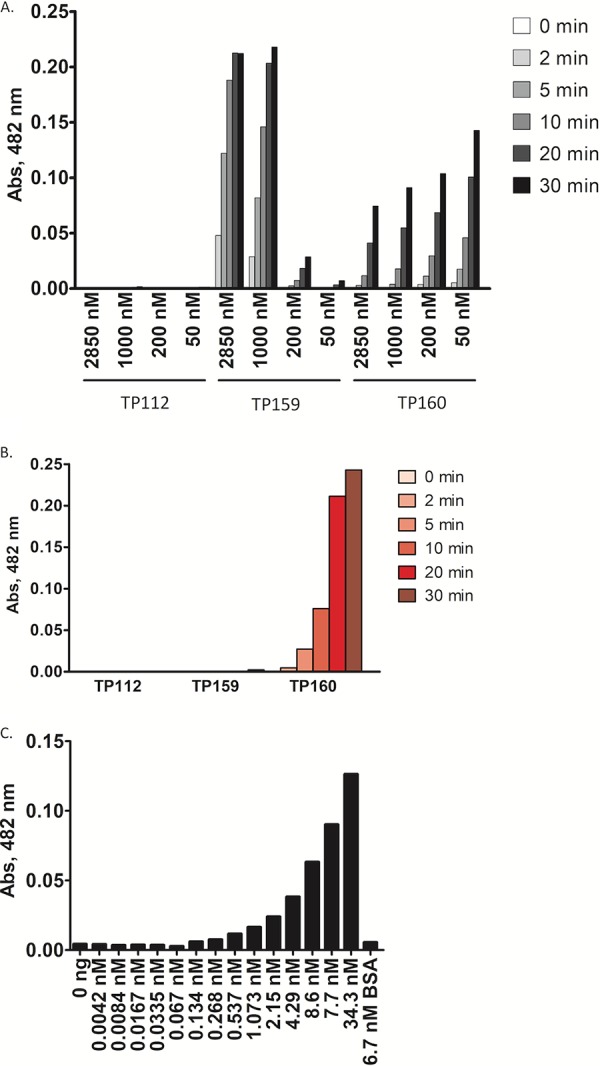
Establishing a KPC-2-specific binding assay. (A) Nitrocefin hydrolysis signals of β-lactamases released from metal affinity resins. Different concentrations of purified BLIP^K74T/W112D^ (50 nM, 100 nM, 200 nM, 1,000 nM, and 2,850 nM) were used to capture β-lactamases from E. coli TP112, TP159, and TP160 cell lysates. Abs, absorbance. (B) Nitrocefin hydrolysis signals of β-lactamases released from metal affinity resins with 10 nM BLIP^K74T/W112D^ added for capture. (C) Nitrocefin hydrolysis signals of KPC-2 at 2 h. A total of 0.125 to 1,000 ng purified KPC-2 was diluted with E. coli cell lysate lacking a β-lactamase and then mixed with 10 nM BLIP^K74T/W112D^.

We released β-lactamases bound to BLIP by adding 30% ethanol, which disrupts the BLIP/β-lactamase interaction but does not affect β-lactamase catalytic activity (23). We simultaneously detected the presence of free β-lactamase by including nitrocefin in the elution buffer and monitoring its hydrolysis (Fig. 3A). No positive signal was observed in the E. coli TP112 (TEM-1) cell lysate, a result consistent with the high *K_i_* value (weak binding) for TEM-1 β-lactamase inhibition by BLIP^K74T/W112D^ (Fig. 2 and Table 1). In contrast, BLIP^K74T/W112D^ efficiently captured KPC-2 from E. coli TP160 cell lysates, as indicated by nitrocefin hydrolysis. Interestingly, the nitrocefin hydrolysis signal increased in the presence of decreasing concentrations of BLIP^K74T/W112D^. At high BLIP^K74T/W112D^ concentrations, the percentage of BLIP^K74T/W112D^/KPC-2 complexes captured by the metal affinity resin was significantly lower than that of free BLIP^K74T/W112D^. This result suggests that, at high concentrations of BLIP^K74T/W112D^, there is more BLIP than β-lactamase and therefore more free BLIP and less BLIP in complex with β-lactamase is captured on the resin when the total BLIP^K74T/W112D^ concentration exceeds the protein binding capacity of the resin. In addition, we observed substantial nitrocefin hydrolysis signal for the E. coli TP159 cell lysate that contains CTX-M-14 β-lactamase (Fig. 3A). This signal can be explained by the expected fractional occupancy of CTX-M-14 by BLIP^K74T/W112D^ of 52% when BLIP^K74T/W112D^ is added to the lysate at 1,000 nM and the expected fractional occupancy of 76% when BLIP^K74T/W112D^ is added at 2,850 nM (Table 2). The fractional occupancy is calculated based on the *K_i_* value of 908 nM for BLIP^K74T/W112D^ inhibition of CTX-M-14 as described in Materials and Methods (34). This signal was significantly reduced when lower concentrations of BLIP^K74T/W112D^ were added to the cell lysate, as predicted by fractional occupancy calculations (Table 2). To further enhance the specificity of BLIP^K74T/W112D^ binding with KPC-2 in cell lysates, the initial BLIP^K74T/W112D^ concentration was decreased to 10 nM when the fractional occupancy of CTX-M-14 is predicted to be 1% while the predicted fractional occupancy of KPC-2 by BLIP^K74T/W112D^ is 81% (Fig. 3B) (Table 2). At the 10 nM BLIP^K74T/W112D^ concentration, the nitrocefin hydrolysis signal from TEM-1 or CTX-M-14 cell lysates was negligible, but KPC-2 detection sensitivity was retained (Fig. 3B).

**TABLE 2:** Calculated fractional occupancy of β-lactamases at various concentrations of BLIP^K74T/W112D^

β-Lactamase	Fractional occupancy (*f*) vs concn of BLIP mutant^*a*^:				
	10 nM	50 nM	200 nM	1,000 nM	2,850 nM
KPC-2	0.806	0.952	0.988	0.998	0.999
CTX-M-14	0.011	0.052	0.182	0.524	0.758
CTX-M-15	0.023	0.103	0.313	0.699	0.870
TEM-1	0.002	0.008	0.031	0.139	0.313

a Fractional occupancy (*f*) is the fraction of β-lactamase bound at each listed concentration of the BLIP^K74T/W112D^ mutant. The equation for determining *f* is provided in Materials and Methods.

To quantify the minimal amount of KPC-2 β-lactamase required in cell lysates for capture and detection using 10 nM BLIP^K74T/W112D^, we performed the KPC-2 binding assay with E. coli cell lysates lacking β-lactamase to which various concentrations of purified KPC-2 β-lactamase were added. The cell lysates were also mixed with bovine serum albumin (BSA) as a negative control. As shown in Fig. 3C, cell lysates with no added KPC-2 or with BSA exhibited limited nitrocefin hydrolysis signal which represents background level. The nitrocefin hydrolysis signal increased in the presence of 3.9 ng to 7.2 ng KPC-2, indicating a low nanogram detection limit for this assay. Taken together, the results establish an *in vitro* binding assay using 10 nM purified BLIP^K74T/W112D^ that detects KPC-2 β-lactamase with 4 ng sensitivity.

### BLIP^K74T/W112D^-based binding assay detects KPC-2 in K. pneumoniae clinical isolates.

**FIG 4:**
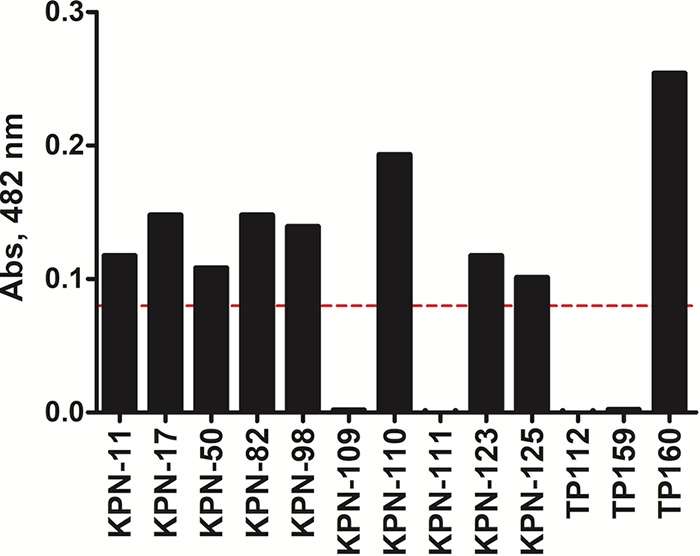
Identification of KPC-2 from K. pneumoniae clinical isolates using the KPC-2-specific binding assay. Nitrocefin hydrolysis signals after BLIP^K74T/W112D^-mediated capture of KPC β-lactamase from cell lysates for the initial 10 K. pneumoniae clinical isolates tested. E. coli TP159 (CTX-M-14) and TP160 (KPC-2) lysates were used as negative and positive controls, respectively. The red dashed line represents the cutoff value for calling a strain KPC positive.

To test the hypothesis that the BLIP^K74T/W112D^-based binding assay accurately identifies clinical strains producing KPC-2, we tested 10 K. pneumoniae isolates that had previously been whole genome sequenced (Materials and Methods) (35). E. coli strains TP159 and TP160, expressing CTX-M-14 and KPC-2, were used as negative and positive controls, respectively. Cell lysate from each strain was used in the binding assay, and 10 nM BLIP^K74T/W112D^ was added to bind KPC-2. BLIP^K74T/W112D^ was captured via its His tag using a metal affinity resin. KPC-2 bound to captured BLIP^K74T/W112D^ was eluted with 30% ethanol and monitored by nitrocefin hydrolysis as described above (Fig. 4). The level of nitrocefin hydrolysis chosen as the cutoff at which to call a strain KPC positive was >0.08. This value is approximately 40-fold higher than the average absorbance signal (0.002) obtained from determinations with lysates from the negative-control strain E. coli TP159 (CTX-M-14). Positive signals were observed in cell lysates prepared from K. pneumoniae strains KPN-11, KPC-17, KPN-82, KPC-98, KPN-109, KPC-110, KPC-123, and KPN-125 and E. coli TP160 (KPC-2), indicating the presence of KPC-2 in these strains. In contrast, no nitrocefin hydrolysis occurred in lysates from K. pneumoniae isolates KPN-50 and KPN-111 and E. coli TP159 (CTX-M-14), suggesting the absence of KPC-2. To evaluate the accuracy of the binding assay, we compared the results to predictions from the genome sequencing results (see Table S1 in the supplemental material). The KPC-2 predictions based on the binding assay were 100% concordant with predictions from genome sequencing (Table S1). Despite the fact that most of the K. pneumoniae strains contained more than one type of β-lactamase based on genome sequencing, the BLIP^K74T/W112D^ binding assay specifically identified KPC-2 in cell lysates.

10.1128/mSphere.00918-19.5 TABLE S1 β-Lactamase content of 127 K. pneumoniae clinical isolates based on genome sequencing results. KPC-2-positive strains identified in this study are indicated in red. Download Table S1, DOCX file, 0.05 MB. Copyright © 2020 Lu et al. 2020 Lu et al. This content is distributed under the terms of the Creative Commons Attribution 4.0 International license.

Next, we tested an additional 117 K. pneumoniae clinical isolates using the same procedure described above. The nitrocefin hydrolysis levels shown in Fig. S2 predicted that K. pneumoniae strains KPN-28, KPN-42, KPN-43, KPN-57, KPN-77, KPN-95, KPN-103, KPN-114, KPN-115, KPN-117, KPN-118, KPN-130, KPN-136, KPN-141, KPN-145, KPN-159, KPN-169, KPN-191, KPN-192, KPN-196, KPN-201, KPN-213, and KPN-218 produce KPC-2 β-lactamase. With the exception of KPN-43, these strains were also identified as KPC-2-positive strains based on whole-genome sequencing results (Table S1). Significant nitrocefin hydrolysis signal was observed from strain KPN-43 (see Fig. S2A in the supplemental material), but whole-genome sequencing results predicted that it was a KPC-2-negative strain (Table S1). To identify whether KPN-43 was a false-positive result, we further tested for the presence of KPC-2 by performing colony PCR using strain KPN-43 and KPC-2-positive strains KPN-11 and KPN-98 and KPC-2-negative strain KPN-111. DNA bands corresponding to the KPC-2 gene were obtained using KPN-43, KPN-11, and KPN-98 as the templates, but not KPN-111 (Fig. S1). Therefore, despite the whole-genome sequence predictions, KPN-43 is a KPC-2-expressing clinical isolate. This indicates that the KPC-43 prediction was not a false-positive result and strongly suggests that the BLIP^K74T/W112D^-based KPC binding assay can be used for sensitive and specific identification of KPC-2 β-lactamase from clinical isolates.

10.1128/mSphere.00918-19.1 FIG S1 Colony PCR results. K. pneumoniae strains KPN-43 (lane 1), KPN-186 (lane 2), KPN-11 (lane 3), KPN-111 (lane 4), and KPN-98 (lane 5) were used as DNA template to amplify the KPC-2 gene using PCR. The 1-kb DNA marker (New England Biolabs) is in lane 6. Download FIG S1, TIF file, 0.4 MB. Copyright © 2020 Lu et al. 2020 Lu et al. This content is distributed under the terms of the Creative Commons Attribution 4.0 International license.

10.1128/mSphere.00918-19.2 FIG S2 (A to D) Nitrocefin hydrolysis signals of KPC released after BLIP^K74T/W112D^-mediated capture from lysates of an additional 117 K. pneumoniae clinical isolates. The level of nitrocefin hydrolysis chosen as the cutoff to call a strain KPC positive is >0.08 as described in the text and in the legend to Fig. 4. The hydrolysis signal was measured 1 h after the addition of nitrocefin. Download FIG S2, TIF file, 2.1 MB. Copyright © 2020 Lu et al. 2020 Lu et al. This content is distributed under the terms of the Creative Commons Attribution 4.0 International license.

### Combination of BLIP^K74T/W112D^ binding and inhibition assays improves detection.

By comparing the BLIP^K74T/W112D^ binding results with whole-genome sequencing results (Table S1), all predicted KPC-2-positive strains from a total of 127 K. pneumoniae isolates were identified except for strains KPN-185 and KPN-186. Thus, KPN-185 and KPN-186 were identified as KPC-2-positive strains by whole-genome sequencing but as KPC-2-negative strains by our protein-based binding assay. As noted above, only strains with absorption signals of >0.08 were considered KPC-2 positive in the BLIP^K74T/W112D^ binding assay. The binding assay identified strain KPN-186 as a KPC-2-negative strain with no observed absorption signal (Fig. S2C). We used colony PCR of the KPN-186 strain as the template and verified KPN-186 as KPC-2 negative despite the prediction based on genome sequencing (Fig. S1). KPN-185, on the other hand, exhibited weak nitrocefin hydrolysis with an absorption signal above that observed for the E. coli TP159 negative-control strain but lower than the 0.08 signal cutoff, resulting in some ambiguity in concluding on the presence or absence of KPC-2 based on binding assay results. Therefore, as a secondary screen, we conducted a BLIP^K74T/W112D^-based inhibition assay on cell lysates prepared from clinical isolates with ambiguous results from our binding assay.

**FIG 5:**
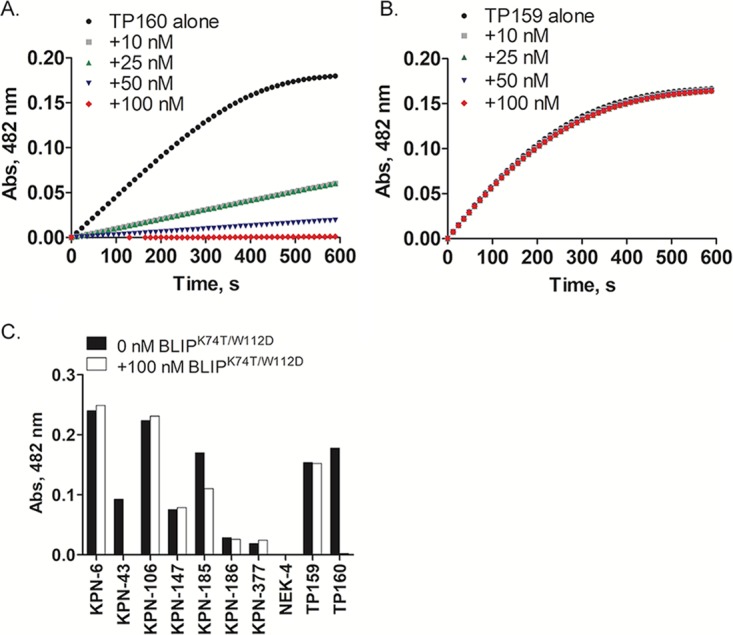
Identification of KPC-2 in K. pneumoniae clinical isolates using the KPC cell lysate inhibition assay. (A) Nitrocefin hydrolysis curves of E. coli TP160 (KPC-2) cell lysate in the presence of increasing concentrations of BLIP^K74T/W112D^; 10 nM, 25 nM, 50 nM, and 100 nM BLIP^K74T/W112D^ were added to TP160 cell lysate. (B) Nitrocefin hydrolysis curves of E. coli TP159 (CTX-M-14) cell lysate in the presence of increasing concentrations of BLIP^K74T/W112D^. (C) Nitrocefin hydrolysis signals from cell lysates of strains KPN-6, KPN-43, KPN-106, KPN-185, KPN-186, KPN-377, KPN-147, NEK-4, TP159, and TP160 in the presence or absence of 100 mM BLIP^K74T/W112D^ at 30 min.

The inhibition assay is based on the observation that cell lysates prepared from KPC-2-expressing strains have nitrocefin hydrolysis activity, which is significantly reduced by the addition of BLIP^K74T/W112D^. To avoid interactions of BLIP^K74T/W112D^ with other β-lactamases in the cell lysate, especially CTX-M-14 and CTX-M-15, we optimized the amount of BLIP^K74T/W112D^ required for the inhibition assay. Various concentrations of BLIP^K74T/W112D^ were added to cell lysates, and β-lactamase activity was monitored by nitrocefin hydrolysis. The presence of BLIP^K74T/W112D^ significantly inhibited the enzymatic activity of KPC-2 in E. coli TP160 cell lysates in a concentration-dependent manner (Fig. 5A). In contrast, CTX-M-14 was not inhibited by BLIP^K74T/W112D^ in E. coli TP159 cell lysates at concentrations up to 100 nM (Fig. 5B). Therefore, 100 nM BLIP^K74T/W112D^ was used in the assay to obtain maximal inhibition of KPC-2.

We conducted the inhibition assay with cell lysates from K. pneumoniae strain KPN-185 and the E. coli TP159 and TP160 control strains in the presence or absence of BLIP^K74T/W112D^. The nitrocefin hydrolysis absorption signals at 30 min are shown in Fig. 5C. In the absence of BLIP^K74T/W112D^, cell lysates prepared from strains KPN-185, TP159, and TP160 all exhibited nitrocefin hydrolysis. Upon the addition of BLIP^K74T/W112D^, a significant reduction in nitrocefin hydrolysis was observed for the KPN-185 (30%) and positive-control E. coli TP160 (100%) cell lysates, indicating KPC-2 β-lactamase is produced in strain KPN-185. Therefore, the inhibition assay successfully identified KPN-185 as a KPC-2-expressing clinical isolate and can be used as a secondary screen for clinical isolates with ambiguous results from the BLIP^K74T/W112D^ binding assay. Hence, the combination of the binding assay results and the inhibition assay results lead to agreement between predictions based on genome sequencing results and also correctly identified KPN-43 as a false-negative result and KPN-186 as a false-positive result from genome sequencing.

In addition to KPN-185, five K. pneumoniae strains, including KPN-6, KPN-106, KPN-147, KPN-377, and NEK-4 (Fig. S2A to D), had absorbance signals from the BLIP^K74T/W112D^ binding assay that were greater than that observed for the E. coli TP159 negative control but less than the 0.08 cutoff. These five strains were classified as KPC-negative strains, which agreed with the predictions based on the whole-genome sequencing data. However, because of the ambiguous signal, we performed the cell lysate inhibition assay on these strains to further verify the presence or absence of KPC-2. All cell lysates conferred nitrocefin hydrolysis activity except for strain NEK-4. The addition of 100 nM BLIP^K74T/W112D^ had no inhibitory effect on lysates for any of the five strains (Fig. 5C), strongly supporting the conclusion that KPC-2 is not produced in these strains. In addition, a complete reduction in nitrocefin hydrolysis signal was observed from the KPN-43 cell lysate in the presence of 100 nM BLIP^K74T/W112D^, while no inhibition was observed for the KPN-186 cell lysate (Fig. 5C). These results further support the predictions based on the BLIP^K74T/W112D^ binding assay that KPN-43 is a KPC-2-positive strain and KPN-186 is a KPC-2-negative strain. It is clear that the BLIP^K74T/W112D^-based cell lysate inhibition assay is a sensitive secondary assay to further verify KPC-2-expressing strains, especially when weak KPC-2 binding signals are observed in the BLIP^K74T/W112D^-based binding assay (Fig. S3).

10.1128/mSphere.00918-19.3 FIG S3 Cell lysate inhibition assay of K. pneumoniae clinical isolates. Nitrocefin hydrolysis signals from cell lysates of 127 K. pneumoniae clinical strains in the absence of BLIP^K74T/W112D^ (black bar) and in the presence of 100 nM BLIP^K74T/W112D^ (white bar) are plotted as a function of time. Download FIG S3, TIF file, 2.9 MB. Copyright © 2020 Lu et al. 2020 Lu et al. This content is distributed under the terms of the Creative Commons Attribution 4.0 International license.

To further verify the results, meropenem susceptibility assays were performed on the 127 K. pneumoniae strains using the carbapenem inactivation method as described in Materials and Methods (18). The 32 strains identified by the BLIP^K74T/W112D^ binding and inhibition assays were also not susceptible to meropenem, as predicted based on the presence of KPC-2 (Table S1). The assays also identified KPN-43 as not susceptible and KPN-186 as susceptible, consistent with the BLIP^K74T/W112D^ binding and inhibition results.

Taken together, the results demonstrate that the BLIP^K74T/W112D^ protein is an effective sensor for KPC β-lactamases and constitutes a KPC-specific detection assay with 100% sensitivity and specificity when using a combination of the BLIP^K74T/W112D^-based binding assay and the BLIP^K74T/W112D^-based inhibition assay.

### Use of BLIP^K74T/W112D^ in the Carba-NP assay identifies KPC in clinical isolates.

A potential limitation of the BLIP^K74T/W112D^-mediated β-lactamase capture assay described above is the need for overnight growth, creation of cell lysates, and manipulation of the affinity resin. The Carba-NP assay is commonly used to detect carbapenemase-producing strains (20). It entails inoculation of colonies to create a suspension, lysis of the cells, and addition of imipenem and the pH indicator molecule phenol red. Hydrolysis of imipenem by a carbapenemase lowers the pH of the solution, which is visually detected as a change in color of the phenol red from red to yellow (20). The BLIP^K74T/W112D^ inhibitor was adapted to the Carba-NP assay by testing colony suspensions in the presence and absence of the inhibitor. A sample that turns yellow in the absence of BLIP^K74T/W112D^ but remains red in the presence of inhibitor indicates the strain produces KPC β-lactamase.

**FIG 6:**
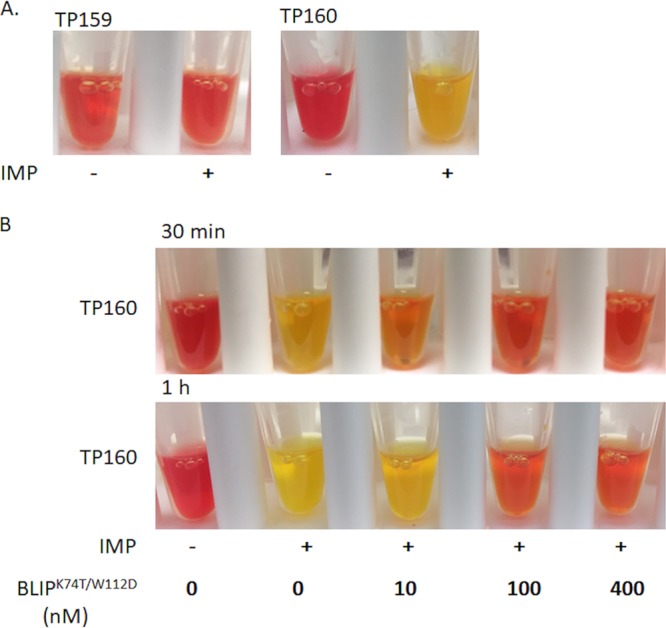
Identification of KPC-2 in K. pneumoniae clinical isolates using the modified Carba-NP test. (A) Carba-NP test results for E. coli TP159 (CTX-M-14) (left) and TP160 (KPC-2) (right). Phenol red with (+) or without (−) 6 mg/ml imipenem (IMP) was mixed with cell lysate and incubated at 37°C for 1 h. (B) Modified Carba-NP test results for E. coli TP160 (KPC-2) in the presence of BLIP^K74T/W112D^. Different concentrations of BLIP^K74T/W112D^ (0, 10, 100, and 400 nM) were added to cell lysates and incubated at room temperature for 10 min.

The Carba-NP assay with BLIP^K74T/W112D^ was initially tested with the E. coli TP159 and TP160 strains producing CTX-M-14 and KPC-2 β-lactamases. For this purpose, a loop of cells was added to a solution containing lysozyme and detergent for cell lysis, imipenem, phenol red, and the presence or absence of BLIP^K74T/W112D^ (Materials and Methods). Cell suspensions from E. coli TP159 producing CTX-M-14 remained red with or without the addition of BLIP^K74T/W112D^ (Fig. 6). In contrast, cell suspensions from E. coli TP160 producing KPC-2 turned yellow in the absence of BLIP^K74T/W112D^ but remained red when the inhibitor was present. These results suggest that the assay can be used to identify KPC-producing strains (Fig. 6B). In addition, the E. coli TP160 strain was used to show that imipenem is required for the color change and that the addition of 200 nM BLIP^K74T/W112D^ is sufficient to block the color change (Fig. 6B).

We next performed the Carba-NP assay with BLIP^K74T/W112D^ and the 127 K. pneumoniae clinical isolates described above. The results obtained were consistent with the results of the BLIP^K74T/W112D^ binding and inhibition assays (Fig. S4). Strains KPN-11, KPC-17, KPN-28, KPN-42, KPN-43, KPN-57, KPN-77, KPN-82, KPN-95, KPC-98, KPN-103, KPN-109, KPC-110, KPN-114, KPN-115, KPN-117, KPN-118, KPC-123, KPN-125, KPN-130, KPN-136, KPN-141, KPN-145, KPN-159, KPN-169, KPN-185, KPN-191, KPN-192, KPN-196, KPN-201, KPN-213, and KPN-218 were predicted to be KPC-2-positive strains (Fig. S4). These strains were also identified as KPC-2-positive strains based on whole-genome sequencing results and the binding and cell lysate inhibition assays described above (Table S1). Note that we were only able to confirm strain KPN-185 as KPC-2 positive and strains KPN-6, KPN-106, KPN-147, KPN-377, and NEK-4 as KPC-2 negative using the combination of the binding and cell lysate inhibition assays (Fig. S2 and Fig. 5C). In contrast, with the Carba-NP assay, color differences for phenol red in the presence and absence of BLIP^K74T/W112D^ for strain KPN-185 were apparent (Fig. S4). As expected based on genome sequencing results, no color changes occurred for strains KPN-6, KPN-106, KPN-147, KPN-377, and NEK-4 due to lack of carbapenemase activity.

10.1128/mSphere.00918-19.4 FIG S4 Carba-NP assay with BLIP^K74T/W112D^ of Klebsiella pneumoniae clinical isolates. The strain names are above each panel and correspond to those shown in Table S1. Tubes a contain K. pneumoniae lysate with phenol red. Tubes b contain imipenem and phenol red. Yellow color formation in tube b indicates the presence of a carbapenemase. Tubes c contain imipenem, phenol red, and 200 nM BLIP^K74T/W112D^. If tubes b and c are yellow, a carbapenemase that is not KPC is present. If tube b is yellow and tube c is red, a KPC carbapenemase is present. Download FIG S4, TIF file, 2.5 MB. Copyright © 2020 Lu et al. 2020 Lu et al. This content is distributed under the terms of the Creative Commons Attribution 4.0 International license.

The Carba-NP assay with BLIP^K74T/W112D^ was also performed with K. pneumoniae strains known to produce either the NDM-1 metallo-β-lactamase or the OXA-48 carbapenemase. The assay revealed NDM-1 or OXA-48 production based on color change of the suspension from red to yellow. However, addition of BLIP^K74T/W112D^ did not alter the color reaction, a result indicating the absence of KPC β-lactamase. This result shows that BLIP^K74T/W112D^ does not inhibit NDM-1 or OXA-48, as predicted based on the *in vitro* inhibition assays (Table 1 and Fig. S4). Therefore, the Carba-NP assay coupled with BLIP^K74T/W112D^ can readily identify KPC-producing strains and can distinguish strains producing KPC from those producing other carbapenemases.

## DISCUSSION

Carbapenems are critical agents used to treat serious infections caused by Gram-negative bacteria. Unfortunately, many bacterial pathogens have developed resistance to carbapenems by producing β-lactamases such as the KPC β-lactamases (8, 36). In principle, when KPC is associated with a bacterial infection, effective β-lactams or antimicrobial combinations such as ceftazidime-avibactam and meropenem-vaborbactam can be used to kill the pathogen (37, 38). Thus, rapid identification of KPC in clinical isolates will facilitate the administration of correct treatment regimens for carbapenem-resistant infections, thereby decreasing patient morbidity and mortality.

In this work, we report development of a β-lactamase binding and inhibition assay that specifically identifies KPC carbapenemases made by clinical isolates. For our binding and inhibition assay, we chose a previously characterized BLIP^K74T/W112D^ variant as the KPC sensor (23). We showed that BLIP^K74T/W112D^ specifically binds and inhibits the enzyme activity of KPC-2 and KPC variants, but not other β-lactamases (Table 1). In addition, BLIP is an easily purified, soluble protein, with a relatively low molecular weight (17.5 kDa) and is stable at room temperature. Hence, BLIP^K74T/W112D^ is a good candidate for use in the recognition of KPCs *in vitro*.

The BLIP^K74T/W112D^ binding and inhibition assay as performed here involves growing a culture overnight and using 1 ml for cell lysis with 1% Triton X-100. The lysate was cleared by centrifugation and incubated for 1 h with commercially available Talon beads and washed four times before the addition of 30% ethanol with nitrocefin. The total time to process after overnight growth, including incubation time, is approximately 3 h. However, shorter incubation times are possible, and optimization is in process.

Here, we showed that the minimum amount of KPC-2 that can be detected in the binding assay is 4 ng. KPC-2 has a molecular weight of ∼29,000 g/mol, and therefore, 4 ng of KPC-2 equals approximately 0.14 pmol or 10^11^ molecules in 1 ml lysis buffer. The overnight cultures of K. pneumoniae contain ∼3 × 10^9^ cells per ml. Therefore, a KPC-2 expression level of approximately 40 molecules per cell in the 1 ml of culture used is required to produce 4 ng of KPC-2. A previous study by Soufi et al. demonstrated that the dynamic range of protein copy numbers is approximately 1 to 300,000 protein molecules per bacterial cell (39). The majority of proteins are expressed at copy numbers between 10^2^ and 10^4^, which are significantly larger values than the 40 protein molecules per cell required to detect KPC-2. Thus, the binding assay should detect KPC-2 from K. pneumoniae with lower than average expression levels for bacteria.

An interesting observation from the cell lysate inhibition assay is that nitrocefin hydrolysis was decreased for cell lysates in the presence of 100 nM BLIP^K74T/W112D^ for 29 of 32 strains (see Fig. S3 in the supplemental material). This finding suggests that KPC-2 is the dominantly expressed β-lactamase in KPC-2-positive strains despite the presence of genes for other β-lactamases. Although this conclusion is based on analysis of only 127 clinical isolates, if broadly true, it further supports the value of the assay. As discussed above, KPC-2 has a broader spectrum of β-lactam and inhibitor resistance compared to the majority of other β-lactamases. If KPC-2 is the dominantly expressed β-lactamase in its bacterial host strain, identification of KPC-2 will be an effective strategy to inform an antibiotic treatment plan in that antibiotics such as ceftazidime-avibactam and meropenem-vaborbactam are effective against KPC-producing organisms.

The Carba-NP assay is a rapid assay to detect carbapenemase-producing bacteria. Modifications of the assay, such as the Carba-NP-II method where the inhibitors tazobactam and EDTA are added in parallel with the standard assay to distinguish the molecular class of carbapenemase present, i.e., Ambler class A, B, or D (40, 41). The modified test, however, does not indicate the specific carbapenemase present in the strain.

We explored the possibility of using the BLIP^K74T/W112D^ protein in conjunction with the Carba-NP phenotypic test. We showed that performing the Carba-NP test with or without BLIP^K74T/W112D^ can be used to detect a carbapenemase and determine whether it is a KPC β-lactamase. Thus, while it should be possible to optimize the time involved in the BLIP^K74T/W112D^ binding and inhibition assay, the BLIP^K74T/W112D^ protein can be used in the rapid Carba-NP assay to identify KPC β-lactamase.

In conclusion, we have shown that an engineered BLIP^K74T/W112D^ protein displays highly specific KPC-2 binding and provides a 100% identification rate of KPC-2 from clinical isolates. Because all KPC variants tested in this study had tight binding affinity toward BLIP^K74T/W112D^, the data strongly support its use as a sensor for the detection of KPC β-lactamases from clinical isolates.

## MATERIALS AND METHODS

### Strains and plasmids.

Plasmid pGR32 with the BLIP^K74T/W112D^ gene (23) was transformed into E. coli RB791 (W3110 *lacI*^q^L8) (42) for BLIP production and purification.

The variant KPC genes were inserted into plasmid pET-28a^+^ with an N-terminal 6×His tag. The resulting plasmids were transformed into E. coli BL21(DE3) {*fhuA2* [*lon*] *ompTgal* (λ*DE3*) [*dcm*] Δ*hsdS* λ*DE3 =* λ *sBamHIo* Δ*EcoRI-B int*::(*lacI::Plac*UV5::*T7gene1*) *i21* Δ*nin*5} for protein production and purification (43).

E. coli TP112 {F^−^ *mcrA* Δ(*mrr-hsdRMS*-*mcrBC*) ϕ80d*lac*Z M15 Δ*lacX74 deoR recA1 endA1 araD139* Δ(*ara leu*)*7649 galU galK rspL nupG pyrF*::*bla*_TEM-1_ [λ*cI*857 (*cro-bioA*) *tet*]} (28), TP159 {F^−^ *mcrA* Δ(*mrr*-*hsdRMS*-*mcrBC*) ϕ80d*lac*Z M15 Δ*lacX74 deoR recA1 endA1 araD139* Δ(*ara leu*)*7649 galU galK rspL nupG pyrF*::*bla*_CTX-M-14_ [λ*cI*857 (*cro-bioA*) *tet*]}, and TP160 {F^−^ *mcrA* Δ(*mrr*-*hsdRMS*-*mcrBC*) ϕ80d*lac*Z M15 Δ*lacX74 deoR recA1 endA1 araD139* Δ (*ara leu*)*7649 galU galK rspL nupG pyrF*::*bla*_KPC-2_ [λ*cI*857 (*cro-bio*A) *tet*]} (23) express the TEM-1, CTX-M-14, and KPC-2 enzymes, respectively, from chromosomally located genes. These strains were tested in the β-lactamase binding assay and the cell lysate inhibition assay. E. coli MG1655 (F^−^ λ^−^ *ilvG rfb-50 rph-1*) was used as a β-lactamase negative-control strain.

A total of 127 clinical isolates of Klebsiella pneumoniae isolated from patients in the Houston Methodist Hospital System and characterized by whole-genome sequencing were used to test the sensitivity and specificity of the BLIP assay (35). These 127 strains were chosen from a set of 1,777 K. pneumoniae clinical isolates that had been genome sequenced. The sequencing and strains were extensively described by Long et al. (35). Antibiotic susceptibility data are available for all of the strains. Of the 1,777 K. pneumoniae strains, 581 contained KPC based on genome sequencing data. Of these, 572 (98.4%) were KPC-2 (35). From this collection, a mix of strains was chosen that included those producing KPC-2 based on sequence annotation and those that did not. In addition to the 127 strains described above, 5 strains producing NDM-1 and/or OXA-48 were also chosen from the collection to test the specificity of the Carba-NP/BLIP^K74T/W112D^ assay. Each strain was streaked onto blood agar plates and incubated at 37°C for 24 h. Four or five colonies from each strain were inoculated and grown in Luria-Bertani medium (LB) at 37°C overnight.

### Carbapenem inactivation method.

To verify the carbapenemase susceptibility of K. pneumoniae clinical isolates, we performed previously described carbapenem inactivation methods with modifications (18). Briefly, 1-μl inoculation loop of bacteria was added to 1 ml tryptic soy broth (TSB) (BD BBL tryptic soy broth) and vortexed for 15 s to resuspend the cell pellet thoroughly. Subsequently, a 10-μg meropenem (MEM) disk (BD BBL Sensi-Disc susceptibility test disc) was immersed in the suspension and incubated at 37°C for 4 h. At the end of the incubation period, a freshly inoculated E. coli ATCC 25922 strain (a carbapenem-susceptible strain) with a turbidity equivalent to a McFarland value of 0.5 was streaked in three directions using a sterile cotton swab onto a Mueller-Hinton plate (BD DDL Mueller-Hinton agar). The MEM disk was then removed from the suspension using a 10-μl inoculation loop and placed onto the Mueller-Hinton plate. After incubation at 37°C overnight, meropenem susceptibility was determined by direct measurement of inhibition zone diameter. Strains with an inhibition zone diameter greater than 20 mm were classified as meropenem susceptible.

### Protein production and purification.

BLIP^K74T/W112D^ containing an N-terminal 6×His tag was purified from E. coli strain RB791 as previously described with modifications (23). E. coli cells with plasmid pGR32-BLIP^K74T/W112D^ were cultured in LB medium at 37°C, and protein expression was induced with 6 mM d-lactose for 26 h at 23°C when the optical density at 600 nm (OD_600_) reached 0.9 to 1.0. Cells were harvested the next day by low-speed centrifugation and suspended in lysis buffer containing 20 mM Tris-HCl (pH 8.0), 500 mM NaCl, and Xpert protease inhibitor cocktail (GenDEPOT). Cells were sonicated, and cell debris was removed by centrifugation at 8,000 × *g* at 4°C for 20 min. The supernatant was loaded onto a column containing Co^2+^-charged Talon resin and the flow through was collected. This step was repeated once to increase protein binding. After washing, BLIP^K74T/W112D^ was eluted with lysis buffer supplemented with 400 mM imidazole. To further purify BLIP^K74T/W112D^, the eluted protein fractions were concentrated and loaded onto a Superdex75 Increase 10/300 gel filtration column (GE Healthcare) equilibrated with 20 mM Tris-HCl (pH 8.0) and 200 mM NaCl buffer. Protein purity was assessed with sodium dodecyl sulfate-polyacrylamide gel electrophoresis (SDS-PAGE), followed by Coomassie brilliant blue (CBB) staining.

For purification of KPC-2 and variant enzymes, E. coli BL21(DE3) cells with plasmid KPC-pET28a^+^ were grown at 37°C to an OD_600_ of ∼1.0 and induced with 0.5 mM IPTG (isopropyl-β-d-1-thiogalactopyranoside) for 20 h at 23°C. Cell pellets were suspended in lysis buffer with 20 mM HEPES-KOH (pH 7.4), 500 mM NaCl, 20 mM imidazole, and Xpert protease inhibitor cocktail and sonicated several times to disrupt cells. Cell debris was removed by centrifugation at 8,000 × *g* at 4°C for 20 min. The supernatant was loaded onto a Co^2+^-charged Talon resin column to allow protein binding. KPC was eluted by lysis buffer supplemented with 40 mM, 100 mM, 250 mM, and 500 mM imidazole, and KPC enzymes were analyzed by SDS-PAGE. KPC-containing fractions were combined, concentrated, and loaded onto a Superdex75 Increase 10/300 gel filtration column in 20 mM HEPES-KOH (pH 7.4) and 200 mM NaCl buffer to further purify the enzymes. Protein purity was assessed by SDS-PAGE, followed by Coomassie brilliant blue staining.

### Enzyme inhibition assays and *K_i_* value determinations.

To determine *K_i_* values of BLIP^K74T/W112D^ against various β-lactamases, enzyme inhibition assays were conducted by measuring hydrolysis of nitrocefin, a colorimetric β-lactam substrate, using a spectrophotometric assay (23). For each assay, a β-lactamase (1 nM KPC-2, 1 nM KPC-3, 1 nM KPC-4, 1 nM KPC-5, 1 nM KPC-6, 1 nM KPC-7, 1 nM KPC-8, 1 nM KPC-9, 0.2 nM CTX-M-14, 0.5 nM CTX-M-15, 1 nM NDM-1, 0.5 nM OXA-48, or 0.375 nM TEM-1) was mixed with increasing concentrations of BLIP^K74T/W112D^ in buffer containing 50 mM sodium phosphate (pH 7.0) and 100 μg/ml bovine serum albumin (BSA) and incubated at room temperature for 20 min. Hydrolysis of nitrocefin was monitored at 482 nm with an Infinite M200 pro plate reader (TECAN). Initial hydrolysis rates of nitrocefin were plotted as a function of BLIP^K74T/W112D^ concentration, and *K_i_* values were obtained by fitting the data to the Morrison tight binding inhibitor equation using GraphPad Prism 5 (44).

The predicted fractional occupancy (*f*) is the fraction of β-lactamase bound to the BLIP^K74T/W112D^ ligand at a given BLIP^K74T/W112D^ concentration, as calculated using the equation *f *= 1/(1 + *K_i_*/[*L*]) where *K_i_* is the inhibition constant and [*L*] is the BLIP^K74T/W112D^ concentration (34).

### BLIP^K74T/W112D^ -β-lactamase binding assay.

To establish an assay for specific detection of KPC-2 β-lactamase, pulldown experiments of KPC-2 were performed with different concentrations of BLIP^K74T/W112D^- and β-lactamase-containing cell lysates. To prepare bacterial cell lysates, β-lactamase-containing E. coli strains TP112 (TEM-1), TP159 (CTX-M-14), and TP160 (KPC-2) were grown at 37°C overnight. One milliliter of each culture was harvested by low-speed centrifugation, suspended with 1 ml of 1% Triton X-100 in Tris-buffered saline (TBS) buffer (10 mM Tris-HCl [pH 7.5], 150 mM NaCl) and rotated at room temperature for 45 min. Cell debris was removed by centrifuging at 10,000 × *g* at room temperature for 20 min. To allow binding of BLIP^K74T/W112D^ to β-lactamases in the cell lysate, purified BLIP^K74T/W112D^ was mixed with 1 ml of cell lysate with final concentrations of 10 nM, 50 nM, 100 nM, 200 nM, 1,000 nM, and 2,850 nM and rotated at room temperature for 1 h. Then, 10 μl of Talon resin preequilibrated with TBS buffer was added to the mixtures and rotated at room temperature for 1 h to isolate free BLIP or BLIP/β-lactamase complexes through binding to the His tag on BLIP. Proteins bound nonspecifically were removed by washing the protein-bound Talon resins four times with 1.5 ml TBS with a 30-min incubation between each wash. To release β-lactamase bound to BLIP^K74T/W112D^, the Talon resins were suspended in TBS buffer supplemented with 30 μM nitrocefin and 30% ethanol. Nitrocefin hydrolysis was monitored simultaneously with a M200 pro plate reader (Tecan). The absorption values at 482 nm at different time points were normalized by subtracting values at the zero time point and plotted versus BLIP^K74T/W112D^ concentrations and cell lysate types.

To determine the detection sensitivity of the assay for binding to KPC-2, the binding assay was conducted as described above. To mimic cell lysate conditions, serial dilutions (0.125 to 1,000 ng) of purified KPC-2 was mixed with 1 ml of E. coli MG1655 cell lysate. The binding capacity of 10 nM BLIP^K74T/W112D^ to KPC-2 in the cell lysate was evaluated based on nitrocefin hydrolysis at 482 nm. As a control, 1,000 ng of BSA was added to the E. coli MG1655 cell lysate.

For clinical isolates, the binding assay was performed as described above. Cell lysates were prepared from overnight cultures, and 10 nM BLIP^K74T/W112D^ was used for detection.

### Cell lysate inhibition assay.

Overnight cultures of E. coli strains TP159 and TP160, producing CTX-M-14 and KPC-2 β-lactamases, respectively, were suspended in 1% Triton X-100 in TBS and lysed by incubating at room temperature for 45 min. Cell lysates were further diluted with TBS. Increasing concentrations of BLIP^K74T/W112D^ (0, 10 nM, 25 nM, 50 nM, and 100 nM final) were mixed with the cell lysate at room temperature for 20 min. Nitrocefin was added to 30 μM, hydrolysis was monitored, and the relative absorption for each BLIP^K74T/W112D^ concentration was plotted as a function of time. For clinical samples, the cell lysate inhibition assay was conducted in the presence of 100 nM BLIP^K74T/W112D^ or buffer as a control.

### Carba-NP test.

The Carba-NP test procedure was adapted from a previous study with modifications (20). Cell lysis buffer was prepared by the addition of lysozyme to a final concentration of 300 μg/ml into B-PER Bacterial Protein Extraction Reagent (Thermo Scientific). One or two full 10-μl inoculation loops of E. coli strains TP159 and TP160 were suspended in 400 μl lysis buffer and incubated at room temperature for 10 min to facilitate cell lysis. Final concentrations of 0, 10, 100, and 400 nM BLIP^K74T/W112D^ were then added to cell lysates and incubated at room temperature for 10 min. Subsequently, 40 μl of phenol red solution at pH 7.8 containing 6 mg/ml imipenem was mixed with 40 μl of cell lysate and incubated at 37°C for 1 h. A phenol red solution with no imipenem was added to cell lysates as a negative control. Visual reading of the color in the tubes was performed to assess for the presence of a carbapenemase and whether it was KPC. For clinical samples, the Carba-NP test was conducted in the presence of 200 nM BLIP^K74T/W112D^ or buffer alone as a control.
