# Supplementary material for: Development and Evaluation of a Novel Protein-Based Assay for Specific Detection of KPC β-Lactamases from Klebsiella pneumoniae Clinical Isolates
Source: mSphere. 2020 Jan 8;5(1):e00918-19. doi: 10.1128/mSphere.00918-19 (PMC6952207; doi:10.1128/mSphere.00918-19)
Supplement: TABLE S1 [file mSphere.00918-19-st001.docx]

**TABLE S1** β-lactamase content of 127 *K. pneumoniae* clinical isolates based on genome sequencing results. KPC-2 positive strains identified in this study are indicated in red.
