# Supplementary figures and images for: Development and Evaluation of a Novel Protein-Based Assay for Specific Detection of KPC β-Lactamases from Klebsiella pneumoniae Clinical Isolates
Source: mSphere. 2020 Jan 8;5(1):e00918-19. doi: 10.1128/mSphere.00918-19 (PMC6952207; doi:10.1128/mSphere.00918-19)

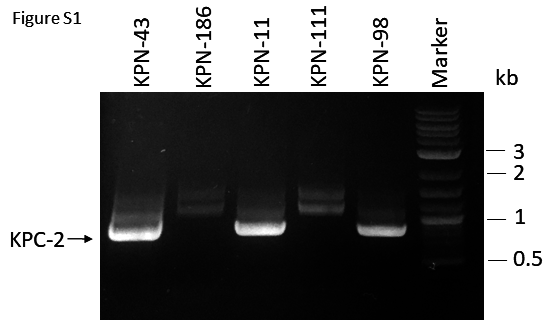

Supplement: FIG S1 [file mSphere.00918-19-sf001.tif]

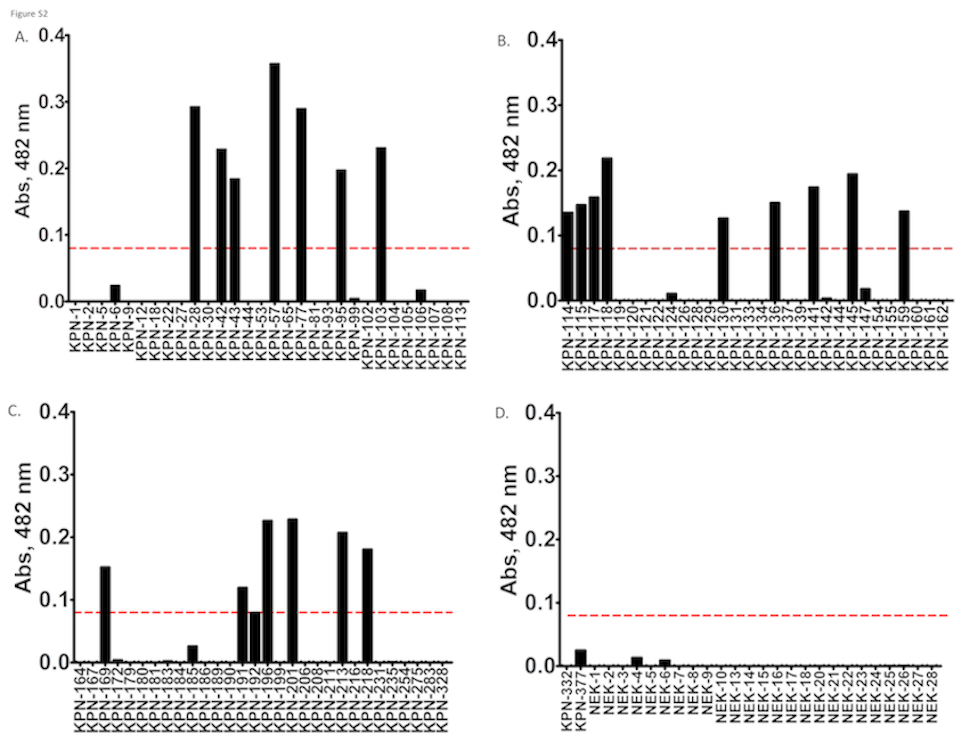

Supplement: FIG S2 [file mSphere.00918-19-sf002.tif]

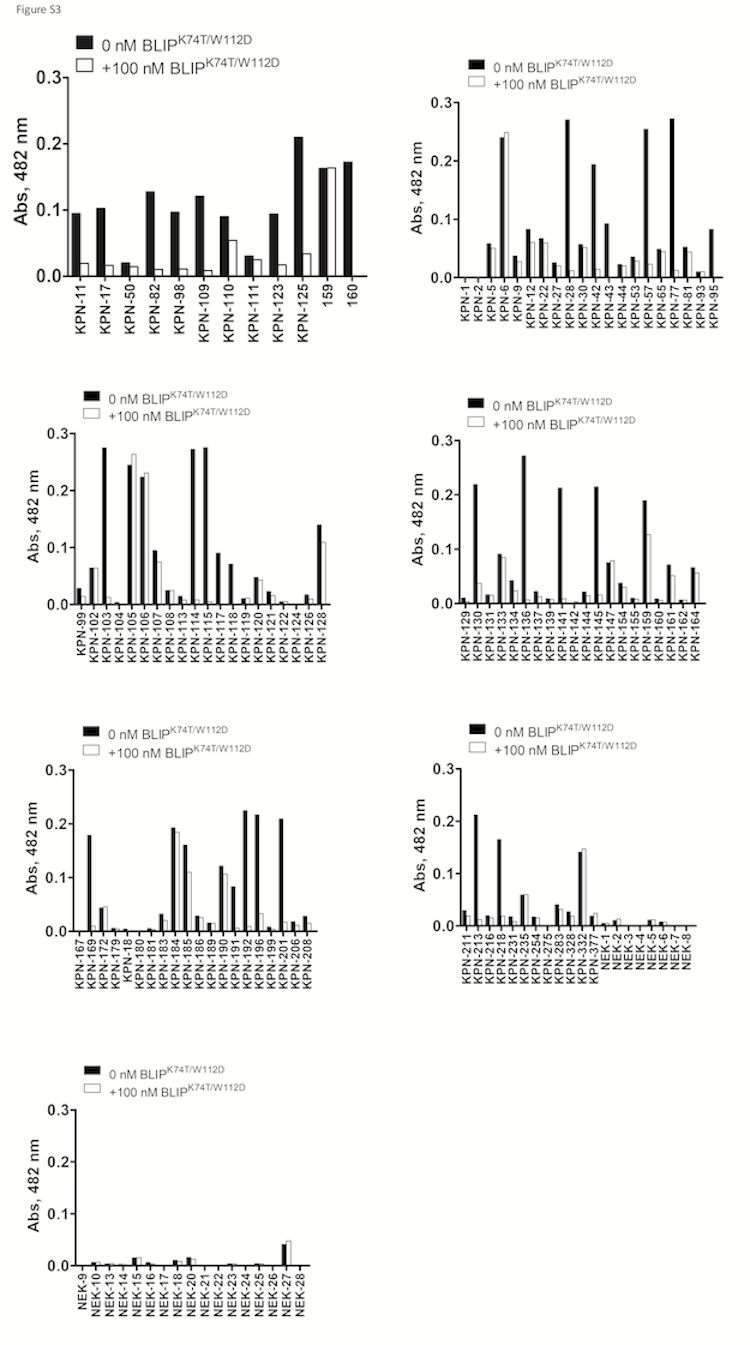

Supplement: FIG S3 [file mSphere.00918-19-sf003.tif]

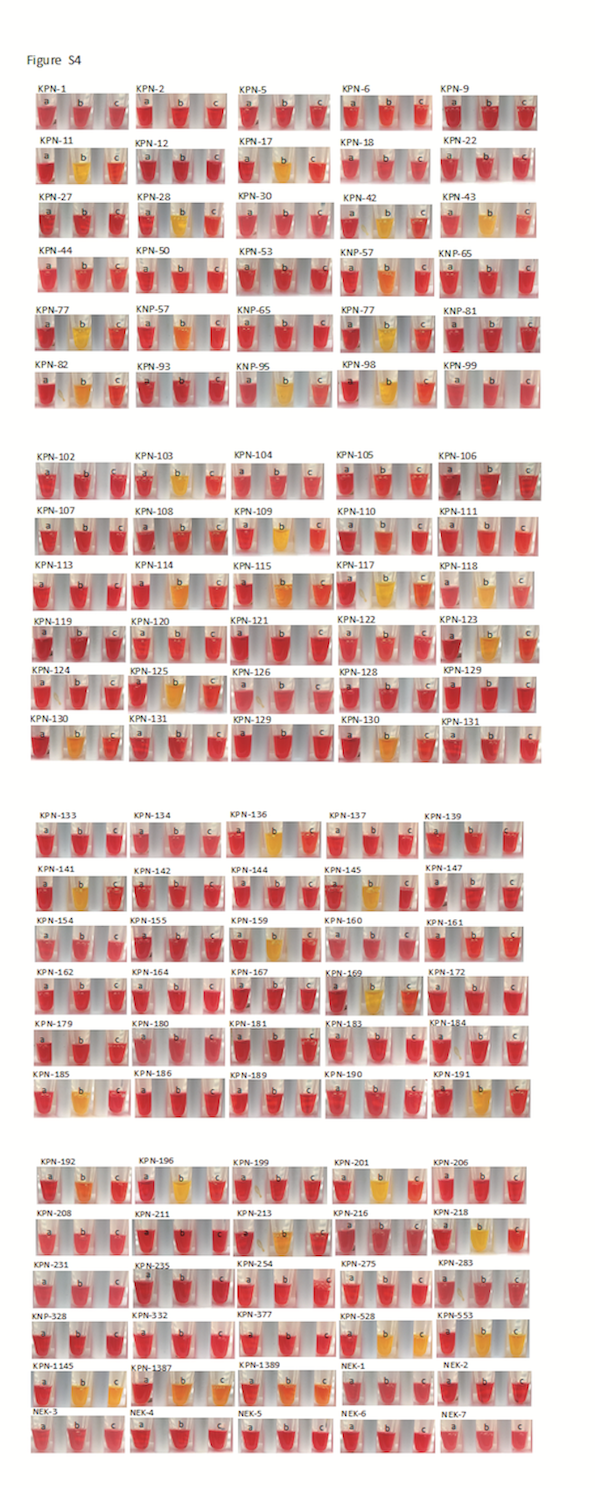

Supplement: FIG S4 [file mSphere.00918-19-sf004.tif]
